# Comparison of Lipoprotein-Associated Phospholipase A2 and High Sensitive C-Reactive Protein as Determinants of Metabolic Syndrome in Subjects without Coronary Heart Disease: In Search of the Best Predictor

**DOI:** 10.1155/2015/934681

**Published:** 2015-05-19

**Authors:** Mónica Acevedo, Paola Varleta, Verónica Kramer, Giovanna Valentino, Teresa Quiroga, Carolina Prieto, Jacqueline Parada, Marcela Adasme, Luisa Briones, Carlos Navarrete

**Affiliations:** ^1^División de Enfermedades Cardiovasculares, Escuela de Medicina, Facultad de Medicina, Pontificia Universidad Católica de Chile, Marcoleta 367, Octavo Piso, Santiago Centro, 8330024 Santiago, Chile; ^2^Unidad de Prevención ACV y Rehabilitación Cardiaca, División de Cardiología y Cirugía Cardiaca, Hospital de la Dirección de Previsión de Carabineros de Chile, Vital Apoquindo 1200, 2°Piso, Las Condes, 7601003 Santiago, Chile; ^3^Departamento de Laboratorios Clínicos, Escuela de Medicina, Facultad de Medicina, Pontificia Universidad Católica de Chile, Avenida Vicuña Mackenna 4686, Macul, 7820436 Santiago, Chile; ^4^Laboratorio Clínico, Hospital de la Dirección de Previsión de Carabineros de Chile, Vital Apoquindo 1200, 2°Piso, Las Condes, 7601003 Santiago, Chile; ^5^Departamento de Matemáticas, Facultad de Ciencias, Universidad de la Serena, Avenida Raúl Bitrán Nachary s/n, 1700000 La Serena, Chile

## Abstract

High sensitivity C-reactive protein (hsCRP) is a marker of metabolic syndrome (MS) and cardiovascular (CV) disease. Lipoprotein-associated phospholipase A2 (Lp-PLA2) also predicts CV disease. There are no reports comparing these markers as predictors of MS. *Methods*. Cross-sectional study comparing Lp-PLA2 and hsCRP as predictors of MS in asymptomatic subjects was carried out; 152 subjects without known atherosclerosis participated. Data were collected on demographics, cardiovascular risk factors, anthropometric and biochemical measurements, and hsCRP and Lp-PLA2 activity levels. A logistic regression analysis was performed with each biomarker and receiver operating characteristic (ROC) curves were constructed for MS. *Results*. Mean age was 46 ± 11 years, and 38% of the subjects had MS. Mean Lp-PLA2 activity was 185 ± 48 nmol/mL/min, and mean hsCRP was 2.1 ± 2.2 mg/L. Subjects with MS had significantly higher levels of Lp-PLA2 (*P* = 0.03) and hsCRP (*P* < 0.0001) than those without MS. ROC curves showed that both markers predicted MS. *Conclusion*. Lp-PLA2 and hsCRP are elevated in subjects with MS. Both biomarkers were independent and significant predictors for MS, emphasizing the role of inflammation in MS. Further research is necessary to determine if inflammation predicts a higher risk for CV events in MS subjects.

## 1. Introduction

Metabolic syndrome (MS) is a group of cardiovascular (CV) risk factors including abdominal obesity, hypertriglyceridemia, low high-density lipoprotein cholesterol (HDL-C), dysglycemia, and high blood pressure (BP) [1]. This condition is associated with a proinflammatory and prothrombotic condition [2]. Numerous studies have shown that a significant increase in systemic inflammatory markers, such as high sensitivity C-reactive protein (hsCRP), interleukin-6, and tumor necrosis factor-*α* (TNF-*α*), among others, is observed in subjects with MS [3].

High sensitivity C-reactive protein is a good predictor of MS and is strongly associated with abdominal obesity [4]. Moreover, this biomarker predicts CV events in healthy people as well as in those with atherosclerotic disease [5, 6]. It has been reported that hsCRP is elevated in an urban population of Santiago, Chile, with MS, and there is a clear correlation between MS, hsCRP, and subclinical atherosclerosis in this population [7]. Thus, the clustering of CV risk factors and inflammation observed in MS increase the risk of atherosclerosis.

Lipoprotein-associated phospholipase A2 (Lp-PLA2) is a recently described inflammatory marker [8]. It is an enzyme produced by macrophages that hydrolyzes phospholipids of oxidized low-density lipoprotein (LDL), releasing oxidized fatty acids and lysophosphatidylcholine, which are potent proinflammatory and prooxidative molecules [8]. Since oxidized LDL is mostly located in the intima of the artery, the products of Lp-PLA2 activity are generated mainly in the vascular wall. Therefore, these mediators play an important role in inflammation at the vascular level. Given that atherosclerosis is an inflammatory disease which begins in the vascular wall, Lp-PLA2 may have a prominent role in its pathophysiology [8].

Both Lp-PLA2 and hsCRP are associated with coronary and cerebrovascular atherosclerotic disease in populations with and without a history of CV events [9–12]. Furthermore, Lp-PLA2 is related to CV risk factors, with a strong correlation with LDL, as well as various components of MS (e.g., abdominal obesity) [13]. The vast majority of published studies do not show any association between Lp-PLA2 and hsCRP; hence, these inflammatory markers have been considered to be independent of each other. There are no reports in literature that compare these markers as predictors of MS.

The objective of this study was to analyze and compare the levels of Lp-PLA2 and hsCRP as predictors of MS in subjects without atherosclerotic disease.

## 2. Materials and Methods

This was a descriptive cross-sectional study in 152 subjects (69 women), without history of atherosclerotic disease, who were recruited in two preventive cardiology centers of Santiago between October 2011 and June 2012: Hospital Clínico de la Pontificia Universidad Católica de Chile and Hospital de la Previsión de Carabineros de Chile. Subjects between 18 and 70 years old were included. Exclusion criteria were (a) history of coronary heart disease and/or carotid or peripheral vascular disease; (b) lipid-lowering therapy (statins, ezetimibe, fibrates, nicotinic acid, and omega 3); (c) use of oral contraceptives or hormone replacement therapy; (d) chronic intake of anti-inflammatory medicines (steroidal and nonsteroidal); (e) acute and/or chronic inflammatory diseases; and (f) pregnancy.

The investigation protocol was approved by the ethics committees of both institutions. Subjects were contacted by telephone and invited to participate by nurses or doctors in charge of the study.

After signing the written, informed consent, patients were subjected to clinical data collection and anthropometric and laboratory measurements, all detailed below.

### 2.1. Data Collection

During the visit to the centers, subjects were interviewed about demographics, medical history, education, physical activity, family medical history, and intake of medications.

#### 2.1.1. Anthropometric and Laboratory Measures

Weight, height, body mass index (BMI), and hip and waist circumferences were measured. The latter was measured in the midpoint between the last rib and the iliac crest. Blood pressure was measured following the recommendations of the Seventh Joint National Committee [14], with the subject sitting and after 5 minutes of rest.

Venous blood samples were taken after 12-hour fasting for determining the following biochemical parameters.

- *Lp-PLA2*: samples for analysis of Lp-PLA2 activity were frozen and stored at −70°C. Levels of Lp-PLA2 activity were determined by enzymatic method (DIADEXUS, USA), Cobas 8000 analyzer, c702 module. Prior to the analysis, precision and trueness of the enzymatic test were assessed locally, and a calibration curve was constructed, which was sent to the international center for approval. Following the approval, the analysis of the subjects' samples was performed.
- *Total cholesterol (total-C)*: colorimetric enzymatic method, Roche Diagnostics Cobas analyzer Cobas 8000, c702 module.
- *HDL-C*: colorimetric homogeneous enzymatic method, Roche Diagnostics Cobas, Cobas 8000 analyzer, c702 module.
- *Triglycerides*: white colorimetric enzymatic method with glycerol, Roche Diagnostics Cobas, Cobas 8000 analyzer, c702 module.
- *LDL cholesterol (LDL-C)*: calculated by Friedewald formula.
- *Blood glucose*: enzymatic method (hexokinase), Roche Diagnostics Cobas, Cobas 8000 analyzer, c702 module.
- *Creatinine*: Jaffé method, Roche Diagnostics Cobas, Cobas 8000 analyzer, c70 module.
- *hsCRP*: nephelometric method on BN ProSpec analyzer, Siemens (detection limit 0.16 mg/L).
- *Fibrinogen*: Clauss method in ACL Top 500 analyzer, Instrumentation Laboratory.

### 2.2. Variables

Hypertensives were subjects who had a prior diagnosis according to the JNC 7 [14], with or without pharmacologic therapy, and/or those with an average blood pressure ≥140/90 mm Hg. Dyslipidemics were subjects who had LDL-C level ≥130 mg/dL, HDL-C level <40 mg/dL in men, <50 mg/dL in women, or non-HDL-C level ≥160 mg/dL in the laboratory assessment. Subjects were considered diabetics if they had a prior diagnosis, with or without drug treatment, and/or if they had a fasting blood glucose ≥126 mg/dL during the study, according to the American Diabetes Association criteria. Current smokers were those subjects who smoked daily during the last month, and ex-smokers were those subjects who had at least six consecutive months without smoking. The recent harmonized criteria were used for the diagnosis of MS, which includes a waist circumference ≥90 cm in men and ≥80 cm in women for Latin American populations [1].

### 2.3. Statistical Analysis

The software R 2.15.2 was used for statistical analysis. A logistic regression analysis was performed with each biomarker, adjusted for age and gender. Smooth receiver operating characteristic (ROC) curves were constructed with cubic splines for MS (area under the curve *C* value = 0.50 implies a predictive value equivalent to chance). Comparisons of means (expressed as mean ± standard deviation [SD]) are based on the analysis of variance and linear regression models.

## 3. Results

This study included 152 subjects (45% women) with a mean age of 46 ± 11 years.  Table 1 shows demographic data and prevalence for CV risk factors. Prevalence rate for hypertension, diabetes, dyslipidemia, and current smoking was 30%, 5%, 62%, and 31%, respectively, with no significant differences observed between genders. Prevalence of MS was 38% (57 subjects) in the total sample with no significant differences between genders: 42% and 32% in men and women, respectively.  Table 2 provides anthropometric and laboratory measures from all subjects divided by gender. Men had significantly higher waist circumference (*P* < 0.01), systolic and diastolic BP (*P* < 0.0001), and creatinine levels (*P* < 0.0001) than women. Women had higher HDL-C levels (*P* < 0.0001).

Mean level of hsCRP was 2.1 ± 2.2 mg/L, and there were no significant differences between men and women (Table 2). Likewise, there were no significant differences in the prevalence of high levels of hsCRP (defined as >2 mg/L) between genders (39% in men and 36% in women). Of note, hsCRP was significantly and directly correlated with BMI, waist circumference, blood glucose, systolic BP, non-HDL-C, and fibrinogen, and it was inversely and significantly correlated with HDL-C (Table 3).

Mean level of Lp-PLA2 activity was 185 ± 48 nmol/mL/min in the total sample with significant differences between men and women: 201 nmol/mL/min and 166 nmol/mL/min (*P* < 0.0001), respectively (Table 2). Lp-PLA2 was significantly and directly correlated with BMI, waist circumference, diastolic BP, LDL-C, non-HDL-C, and plasma creatinine; HDL-C was inversely and significantly correlated with Lp-PLA2. No significant correlations were found between Lp-PLA2 and blood glucose, systolic BP, hsCRP, and fibrinogen (Table 3). No differences were found between Lp-PLA2 activity of active smokers and nonsmokers.

**Figure 1:**
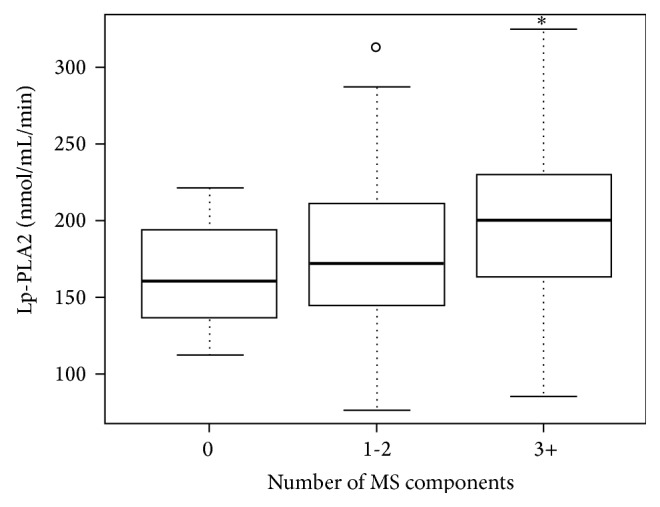
Levels of lipoprotein-associated phospholipase A2 by number of metabolic syndrome components. ^∗^ *P* < 0.01 versus 0 MS components. LpPLA2: lipoprotein-associated phospholipase A2; MS: metabolic syndrome.

**Figure 2:**
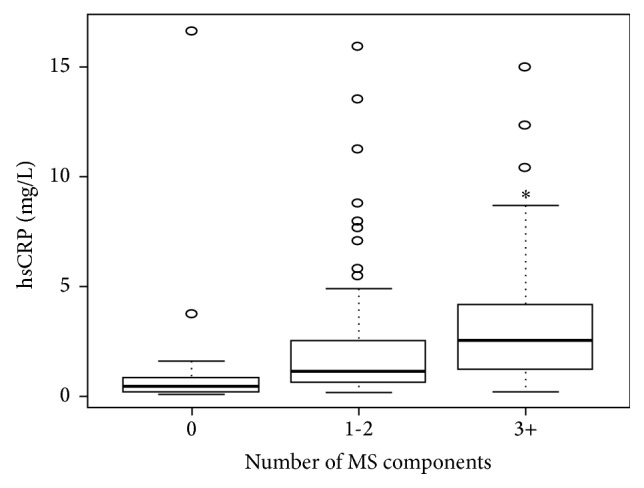
Mean levels of high sensitive C-reactive protein by number of metabolic syndrome components. ^∗^ *P* = 0.04 versus 0 MS components. hsCRP: high sensitivity C-reactive protein; MS: metabolic syndrome.

As shown in Table 4, both Lp-PLA2 and hsCRP were significantly higher in subjects with MS than in those without MS. In subjects with MS, Lp-PLA2 was 198 ± 45 nmol/mL/min versus 180 ± 48 nmol/mL/min in subjects without MS (*P* = 0.03). Similarly, hsCRP was 4.1 ± 3.3 mg/L in subjects with MS versus 2.2 ± 3.2 mg/dL in those without MS (*P* = 0.0001). Both biomarkers significantly increased with the number of MS components: Lp-PLA2 levels increased from 163 nmol/mL/min to 198 nmol/mL/min (*P* < 0.01) and hsCRP increased from 1.5 mg/L to 4.1 mg/L (*P* = 0.04) when subjects with 0 and ≥3 components of MS were compared (Figures 1 and 2).

**Figure 3:**
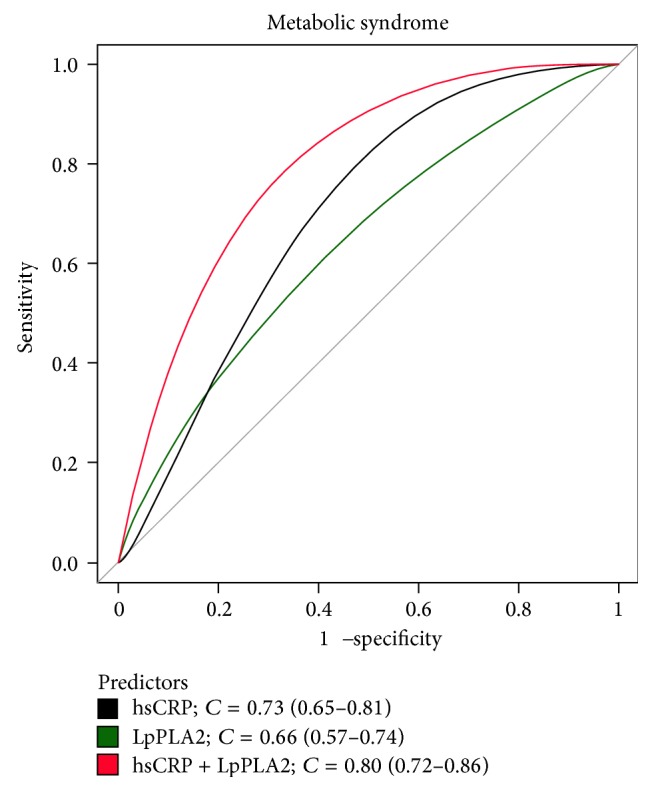
ROC curves for lipoprotein-associated phospholipase A2 (Lp-PLA2), high sensitivity C-reactive protein (hsCRP), and both together adjusted by age and gender.

The results of the logistic regression analysis for determining MS, which included both biomarkers adjusted for age and gender, were as follows: odds ratio (OR) for LpPLA2: 1.02 (confidence interval [CI]: 1.00–1.02, *P* = 0.03) and OR for hsCRP: 2.5 (CI: 1.65–3.80, *P* < 0.0001).  Figure 3 shows the ROC curves for both biomarkers adjusted for age and gender: Lp-PLA2, *C* value = 0.66 [0.57–0.74] and hsCRP, *C* value = 0.73 [0.65–0.81]. According to this analysis, although hsCRP shows a better area under the curve, from a statistical point of view, it cannot be shown that it is better than Lp-PLA2, as the CIs of both biomarkers overlap.

## 4. Discussion

In this study, we have demonstrated in a population without atherosclerotic disease that levels of Lp-PLA2 activity and hsCRP are elevated in subjects with MS. Both biomarkers significantly increased with the number of metabolic risk factors, and both were shown to be independent and statistically significant predictors of MS. These results highlight the role of inflammation in MS.

The MS corresponds to a clustering of CV and metabolic risk factors and inflammation. In our country, MS prevalence is approximately 30%, and it is occurring at younger ages, probably due to the overweight, obesity, and insulin resistance epidemic. Although, the pathophysiologic mechanism involved in MS is not entirely clear, it has been shown that it confers an increased risk for diabetes and atherosclerotic CV disease [9, 15].

Inflammation plays a fundamental role in the development of MS. Recent studies have reported that the presence of MS and inflammation (determined by hsCRP) confers more CV risk than the presence of MS alone [16, 17]. High sensitivity C-reactive protein is also a predictor for the development of MS and diabetes [18]. In our country, we have previously reported that subjects with MS and elevated hsCRP have more subclinical atherosclerosis and carotid atherosclerotic plaques [7].

It is well known that atherosclerosis is an inflammatory disease [19]. Therefore, many inflammatory markers related to MS have been investigated in the search of a possible explanation for the elevated CV risk associated with it. The most studied biomarker is hsCRP; however, there are new biomarkers associated with atherosclerotic disease that may be more specific to vascular inflammation. Among them is Lp-PLA2, an enzyme that acts on oxidized LDL and produces strong inflammatory and oxidative mediators of the intima. Epidemiologic and clinical studies have shown an association between atherosclerotic disease and Lp-PLA2 concentration, in a similar way as reported with hsCRP [9–11, 13].

In this study, we have demonstrated that both biomarkers, Lp-PLA2 and hsCRP, predicted MS with a similar power. Regarding the association of hsCRP with MS, this study confirms the available evidence showing that levels of hsCRP are increased in subjects with MS [16, 17, 20]. Our results also support the association between hsCRP and overall/visceral obesity, HDL-C, non-HDL-C, and glycemia. Similar to the findings of Ballantyne and colleagues [10], we demonstrated that hsCRP is not associated with Lp-PLA2, suggesting that their inflammation pathways are different from a physiologic point of view. Conversely, hsCRP was related to fibrinogen, also an acute phase protein that has a similar mechanism of production by the liver. hsCRP is produced by the liver, primarily by the action of interleukin-6, a cytokine that increases in the conditions of visceral obesity and insulin resistance. Based on this evidence, although hsCRP is an important inflammatory marker, it is unspecific.

Activity levels of Lp-PLA2 were also significantly higher in subjects with MS in our study. These findings are similar to those from the Bruneck Study in 2009 [12]. In that study, Tsimikas et al. found significant associations between Lp-PLA2 and LDL-C, non-HDL-C, HDL-C, and homeostasis model assessment insulin resistance (HOMA-IR), whereas correlations with BMI and waist circumference were weaker. Our results are identical to those reported by these authors regarding lipid factors and obesity. However, we did not find any direct correlation with blood glucose, and we did not measure insulin resistance. Furthermore, Lp-PLA2 was not correlated to BP and smoking in our study, which was seen in the Bruneck Study. Lastly, we found no association between Lp-PLA2 and fibrinogen, which confirms that Lp-PLA2 inflammatory pathway is not through acute phase reaction.

It is important to analyze the relationship between Lp-PLA2 and LDL-C and non-HDL-C (atherogenic cholesterol). After adjusting for gender and age, these correlations became even more significant. Our population had an average LDL-C level of 128 mg/dL, which suggests that, even at moderate levels of LDL-C, subjects with MS had elevated activity of this enzyme. This result could be because many subjects with MS have primarily small and dense LDL molecules, which are prone to oxidation and therefore they are substrate for Lp-PLA2. The inflammation produced by Lp-PLA2 is primarily in the vascular level, because lysophosphatidylcholine and oxidized fatty acids, generated after Lp-PLA2 action over oxidized LDL, induce adhesion molecules and other cytokines in the same vascular wall. Thus, it can be hypothesized that, unlike hsCRP (which occurs as a result of inflammation caused by other cytokines from the visceral fat), Lp-PLA2 is directly involved in the inflammatory process, and, subsequently, the atherogenic process of the plaque. Thus, Lp-PLA2 could be a more specific marker of atherosclerotic events in patients with MS. Confirmation of this hypothesis must be demonstrated by studies of CV events in patients with MS, who have these biomarkers measured, and with findings adjusted for all the risk factors associated with hsCRP and Lp-PLA2.

In this regard, it should be noted that a meta-analysis evaluating the clinical use of hsCRP has shown that this biomarker adds little in the prognosis of CV risk, when adjusting by the risk factors involved in MS [21]. Conversely, the fact that Lp-PLA2 was weakly related to these metabolic factors should preserve its power as a biomarker. However, the latter has not been easy to confirm. The reasons for not having completely cleared Lp-PLA2 prognosis utility are based primarily on the different techniques used for its measurement, which makes it impossible to compare the results of different studies.

In this study, we did not expect to find the same power for hsCRP and Lp-PLA2 as predictors of MS. We expected hsCRP to be a better predictor, given its close association with metabolic risk factors included in MS. However, from a statistical point of view (as demonstrated by the ROC curves), both had a similar level of prediction. These results suggest that Lp-PLA2 is related to MS through other pathways that are not fully known and most likely not associated with the metabolic risk factors. We know that Lp-PLA2 is linked through oxidized LDL, which is elevated in subjects with MS, although it cannot be perceived due to the moderate levels of LDL-C observed in the subjects of our study. The explanation is that Lp-PLA2 is associated with “oxidized” LDL, and, for measuring “oxidized” LDL in the clinic, it required more expensive and sophisticated methods not frequently used in clinical practice.

Since MS is highly prevalent in our population, our results could provide knowledge about nontraditional risk factors that could help to identify which patients with MS must be treated earlier and more aggressively. This is important, because currently there is a pharmacologic inhibitor of Lp-PLA2 activity, darapladib, which is being investigated in subjects with atherosclerotic disease. In addition, another study is currently investigating the use of a monoclonal antibody against interleukin-1 for the intervention against high hsCRP. If these studies have positive results, the measurement and targeting of these biomarkers might become important components of clinical management of patients. Finally, it must be noted that we found no studies in literature that compare Lp-PLA2 and hsCRP as predictors of MS in the general population.

Our study has some limitations: (1) it is a cross-sectional study, and hence causality inferences cannot be made; (2) it was done in a small, nonrandom sample of subjects; (3) we did not include HOMA-IR, which could explain the relationship of insulin resistance with MS and inflammatory markers; (4) finally, we only determined Lp-PLA2 activity instead of mass. This decision was subject to the availability of the laboratory determination in our country. However, currently, it is the most used technique, and it has shown the best correlation with MS [13].

The strengths of the study include the following: (1) the recruitment of subjects was performed prospectively; (2) subjects with lipid-lowering therapy and women with hormone replacement therapy were excluded, among others; (3) this study has validated the measurement of Lp-PLA2 in our country.

In conclusion, Lp-PLA2 and hsCRP are good determinants of MS. Prospective studies must confirm which of the markers or if both markers are causally related to the atherosclerotic consequences observed in subjects with MS.

## Figures and Tables

**Table 1:** Demographic data and prevalence of traditional cardiovascular risk factors in the total sample divided by gender.

	Total (*n* = 152)	Men (*n* = 83)	Women (*n* = 69)	*P*
Age (years)	46 ± 11	45 ± 11	47 ± 11	NS
Educational level (years)	13 ± 3	14 ± 2	13 ± 4	NS
Dyslipidemia (%)	62	68	55	NS
Hypertension (%)	30	31	29	NS
Smoking (%)	31	31	30	NS
Diabetes (%)	5	7	3	NS
Physical inactivity (%)	78	76	80	NS
Overweight (%)	37	41	33	NS
Obesity (%)	33	34	32	NS
Family history of CHD (%)	13	10	16	NS

Data are presented as mean ± SD or percentage.

CHD: coronary heart disease; NS: not significant.

**Table 2:** Anthropometric measurements and laboratory data for all subjects divided by gender.

	Total (*n* = 152)	Men (*n* = 83)	Women (*n* = 69)	*P*
BMI	28 ± 4	28 ± 4	28 ± 5	NS
Waist circumference (cm)	93 ± 11	95 ± 10	90 ± 11	<0.01
Waist ≥ 90/80 cm (men/women), *n* (%)	115 (76%)	61 (74%)	54 (79%)	NS
SBP (mm Hg)	119 ± 15	124 ± 14	113 ± 16	<0.0001
DBP (mm Hg)	76 ± 13	81 ± 11	70 ± 13	<0.0001
Blood glucose (mg/dL)	91 ± 24	93 ± 29	89 ± 16	NS
Total-C (mg/dL)	208 ± 44	209 ± 47	207 ± 39	NS
HDL-C (mg/dL)	52 ± 15	46 ± 13	58 ± 14	<0.0001
LDL-C (mg/dL)	128 ± 36	131 ± 38	126 ± 34	NS
Non-HDL-C (mg/dL)	154 ± 43	158 ± 43	148 ± 42	NS
Creatinine (mg/dL)	0.8 ± 0.2	0.9 ± 0.1	0.7 ± 0.1	<0.0001
hsCRP (mg/L)	2.1 ± 2.2	2.2 ± 2.2	2.0 ± 2.2	NS
Lp-PLA2 (nmol/mL/min)	185 ± 48	201 ± 49	166 ± 38	<0.0001

Values expressed as mean ± SD, except where indicated.

BMI: body mass index; DBP: diastolic blood pressure; HDL-C: high-density lipoprotein cholesterol; hsCRP: high sensitive C-reactive protein; LDL-C: low-density lipoprotein cholesterol; Lp-PLA2: lipoprotein-associated phospholipase A2; NS: not significant; SBP: systolic blood pressure; SD: standard deviation; total-C: total cholesterol.

**Table 3:** Correlation coefficients of Lp-PLA2 with demographic variables, lipid factors, blood glucose, blood pressure, hsCRP, and fibrinogen.

Lp-PLA2			hsCRP		
Variable	Correlation coefficient	*P*	Variable	Correlation coefficient	*P*
BMI	0.20	0.02	BMI	0.54	<0.0001
Waist	0.28	<0.001	Waist	0.48	<0.0001
Blood glucose	0.05	0.56	Blood glucose	0.17	<0.05
SBP	0.09	0.25	SBP	0.23	<0.01
DBP	0.18	0.03	DBP	0.15	0.07
LDL-C	0.62	<0.001	LDL-C	0.15	0.07
HDL-C	−0.45	<0.001	HDL-C	−0.25	<0.01
Non-HDL-C	0.66	<0.001	Non-HDL-C	0.27	<0.001
hsCRP	−0.04	0.67	Lp-PLA2	0.40	<0.0001
Fibrinogen	−0.02	0.79	Fibrinogen	0.40	<0.0001
Creatinine	0.32	<0.001	Creatinine	0.02	NS

BMI: body mass index; DBP: diastolic blood pressure; HDL-C: high-density lipoprotein cholesterol; hsCRP: high sensitive C-reactive protein; LDL-C: low-density lipoprotein cholesterol; Lp-PLA2: lipoprotein-associated phospholipase A2; NS: not significant; SBP: systolic blood pressure.

**Table 4:** Mean levels of hsCRP and Lp-PLA2 by number of metabolic syndrome components according to harmonized criteria^1^ in the total sample.

Total (*n* = 152)	NO MS (0–2 RF) (*n* = 95)	MS (3 or more RF) (*n* = 57)	*P*
LpPLA2 levels (nmol/mL/min)	180 ± 48	198 ± 45	0.03
hsCRP (mg/L)	2.2 ± 3.2	4.1 ± 3.3	<0.0001

hsCRP: high sensitive C-reactive protein; Lp-PLA2: lipoprotein-associated phospholipase A2; MS: metabolic syndrome; RF: risk factors.
